# Heat shock protein 40 of *Streptococcus pneumoniae* induces immune response of human dendritic cells via TLR4‐dependent p38 MAPK and JNK signaling pathways

**DOI:** 10.1002/iid3.735

**Published:** 2022-11-25

**Authors:** Jing‐jing Liu, Jian‐cheng Lin, Li‐na Chen

**Affiliations:** ^1^ Clinical Laboratory Xiamen Children's Hospital Xiamen China

**Keywords:** dendritic cells, Heat shock protein 40, Jun N‐terminal kinase, p38 mitogen‐activated protein kinase, toll‐like receptor 4

## Abstract

**Introduction:**

Heat shock protein 40 (HSP40) is a vaccine adjuvant candidate for *Streptococcus pneumoniae*. The mechanism by which HSP40 activates the human dendritic cells (DCs) is unclear.

**Methods:**

DCs were isolated from human peripheral blood and their markers (HLA‐DR, CD86, CD83, and CD80) were detected by flow cytometry. The messenger *RNA* (mRNA) and secretion levels of inflammary cytokines were measured after DCs were stimulated with recombinant HSP40 (rHSP40). Short hairpin RNAs were used to knock down toll‐like receptor 2 (TLR2) and TLR4. The TLR2‐ or TLR4‐deficient DCs were treated with lipopolysaccharides, rHSP40, or peptidoglycan, and then the secretion levels of tumor necrosis factor‐α (TNF‐α) and interleukin‐6 (IL‐6) were measured. Moreover, the secretion levels of TNF‐α and IL‐6 were measured after DCs were treated with mitogen‐activated protein kinase (MAPK) inhibitors including SB203580, SP600125, and U0126. In addition, the phosphorylation levels of p38 MAPK and Jun N‐terminal kinase (JNK) in DC cells were determined using western blot analysis after treatment with rHSP40 for different times.

**Results:**

DCs were successfully isolated and cultured. rHSP40 treatment significantly increased cytokine levels in a concentration‐dependent manner. TLR4 deficiency, but not TLR2 deficiency, significantly suppressed the rHSP40‐induced secretion of tumor necrosis factor‐α  (TNF‐α) and interleukin‐6 (IL‐6). SB203580 and SP600125 significantly inhibited the rHSP40‐induced secretion of TNF‐α and IL‐6. rHSP40 significantly enhanced the phosphorylation of p38 MAPK and JNK.

**Conclusion:**

HPS40 stimulates the immune response of DCs via the p38 MAPK and JNK signaling pathways, which depend on TLR4.

## Graphical abstract

Activation of dendritic cells (DCs) increases the levels of inflammatory cytokines. The activation of DCs by HSP40 is TLR4‐dependent. HSP40 activates DCs via p38 mitogen‐activated protein kinase (p38 MAPK) and Jun N‐terminal kinase (JNK) signaling pathways.

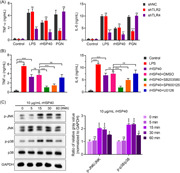

## 1 INTRODUCTION

*Streptococcus pneumoniae*, a gram‐positive bacterium, typically colonizes the human upper respiratory tract. When the body is immunocompromised or infected with viruses, this asymptomatic colonization can develop into invasive diseases such as community‐acquired pneumonia, sepsis, and meningitis.^1^ The elderly population and children are highly susceptible to *S. pneumoniae*, and the resistance of *S. pneumoniae* to antibiotics has gradually increased. Therefore, the exploitation of effective vaccines has become essential to prevent pneumococcal diseases.^2^

Heat shock protein 40 (HSP40), a member of the heat shock protein family, is an important virulence factor of *S. pneumoniae*.^3^ Under stress conditions caused by infection, *S. pneumoniae* highly expresses HSP40 to adapt to external environmental changes.^4^, ^5^ HSP40 is closely related to the immune response, inflammation, and other biological processes.^6^ HSP40 usually participates in the stabilization and maturation of proteins, especially in the aggregation of immune receptors on the cell surface.^7^, ^8^ HSP40 has been chosen as a vaccine adjuvant candidate for *S. pneumoniae*. Previous studies have shown that immunization of mice with HSP40 can induce Th1, Th2, and Th17 immune responses in mice, significantly reduce the colonization of *S. pneumoniae* in the nasal cavity and lungs, and exhibits a protective effect against invasive infection of *S. pneumoniae* with different serotypes.^9^ Moreover, HSP40 also activates mouse macrophages to secrete interleukin‐6 (IL‐6) via phosphatidylinositol 3‐kinase (PI3K) pathway.^3^, ^6^ In addition, HSP40 activates mouse dendritic cells (DCs) derived from bone marrow via Jun N‐terminal kinase (MAPK), nuclear factor kappa B (NF‐κB), and PI3K‐serine/threonine‐protein kinase (PI3K‐Akt) pathway.^10^ However, these findings are all dependent on the experiments conducted in mice.

DCs are the most potent antigen‐presenting cells (APCs), which are important for the initiation of adaptive immune responses and the maintenance of peripheral tolerance.^11^ Upon capturing antigens, DCs undergo maturation. For example, the binding of lipopolysaccharides (LPS) derived from gram‐negative bacteria to toll‐like receptor 4 (TLR4) initiates the activation and maturation of DCs.^12^ Mature DCs have higher expression levels of CD80, CD40, CD83, CD86, major histocompatibility complex I (MHC I), and MHC II (HLA‐DR, HLA‐DP, and HLA‐DQ).^13^, ^14^, ^15^, ^16^ After maturation, DCs enhanced antigen presentation and secreted higher levels of inflammatory cytokines such as IL‐1β, tumor necrosis factor‐α (TNF‐α), IL‐6, IL‐10, IL‐12, and IL‐23, regulating the immune responses.^17^, ^18^, ^19^ This study, for the first time, aims to investigate the activation of human DCs by HSP40.

Previous studies reported that HSP60 and HSP70 are recognized by TLR2 and TLR4,^20^, ^21^, ^22^ whose subsequent downstream signaling triggers MAPK signaling pathways.^23^ MAPK signaling pathway mainly includes four pathways: p38 MAPK pathway, Jun N‐terminal kinase (JNK)/stress‐activated protein kinase (SAPK) pathway, extracellular signal‐regulated protein kinase (ERK) pathway, and big MAP kinase 1 (BMK1)/ERK5 pathway.^24^ MAPK signaling pathway is involved in the immune response induced by *S. pneumoniae*.^25^, ^26^ The p38 MAPK signaling pathway was reported to be activated in human pulmonary epithelial cells when hosts are infected with *S*. *pneumoniae*.^27^ *Streptococcus pneumoniae* also induces JNK‐dependent secretion of IL‐8 in human bronchial epithelial cells.^28^ Thus, we hypothesized that HSP40 may also activate human DCs via MAPK signaling pathway dependent on TLRs.

## 2 MATERIALS AND METHODS

### 2.1 Blood samples

We included healthy individuals between the ages 18 and 60 and excluded those with abnormal results of routine blood tests (including red blood cell count, hemoglobin, white blood cell count, differential white blood cell count, and platelet count), hematologic disorders, or immune‐mediated diseases by interview of the study subject, medical records review, and screening blood tests. Blood samples (100 ml/person) were collected from three human donors. Informed consent was obtained from all donors. This study was approved by the Scientific Research Ethics Committee of the Xiamen Children's Hospital ([2021]NO.40) and conducted in Xiamen Children's Hospital for 1 year.

### 2.2 Guidelines statement

The study was performed in accordance with Declaration of Helsinki.

### 2.3 PBMC isolation and DCs purification

Peripheral blood mononuclear cells (PBMCs) were isolated using Ficoll isolation kit (CAT# LDS1075‐1, TBDsciences) as previously mentioned.^29^ Human DCs were purified from PBMCs using the Human DC Enrichment kit (CAT# 11308D, ThermoFisher Scientific) according to the manufacture's instruction.

### 2.4 Cell staining by flow cytometry

Twenty‐four hours after treatment with 5 μg/ml LPS (CAT# L4391, Sigma‐Aldrich), DCs were harvested and stained with fluorescein isothiocyanate (FITC) anti‐human CD86 (CAT# 374204, Biolegend), FITC anti‐human CD80 (CAT# 375405, BioLegend), FITC anti‐human HLA‐DR (CAT# 327005, Biolegend), and FITC anti‐human CD83 (CAT# 305305, BioLegend) at 28°C for 10 min. After washing with 0.5% bovine serum albumin (BSA; CAT# 4240, BioFroxx) in phosphate‐buffered saline (PBS; CAT# IMMOCELL, IMMOCELL), the stained cells were analyzed using a flow cytometer NovoCyte 1300 (ACEA) within the FITC channel (Ex: 488 nm/Em: 519 nm).

### 2.5 Expression and purification of recombinant HSP40 (rHSP40)

rHSP40 was supplied by DETAIBIO (Nanjing). Briefly, the complementary DNA (cDNA) of *HSP40* was synthesized and cloned into the pET‐30a vector. The plasmid pET‐30a‐rHSP40 was transfected into *Escherichia coli* BL21 (DE3) to express rHSP40. rHSP40 was purified using a Ni‐NTA Purification System (ThermoFisher Scientific).^30^ Residual LPS was detected using an endotoxin detection kit (CAT# EC80545, BIOENDO) according to the manufacture's instruction. The concentration of rHSP40 was detected using a Detergent Compatible Bradford Protein Assay kit (CAT# P0006C, Beyotime).

### 2.6 DCs culture and treatment

DCs were cultured and processed as previously described.^31^ DCs were cultured in GT‐T505 medium (Takara) supplemented with 50 ng/ml IL‐4 (CAT# GMP‐TL104, T&L Biological Technology) and 100 ng/ml granulocyte‐macrophage colony‐stimulating factor (GM‐CSF; CAT# GMP‐TL302, T&L Biological Technology) at 37°C in a 5% CO_2_ incubator. During stimulation, DCs were seeded into six‐well plates at a density of 3 × 10^6^ cells per well and treated with 5 μg/ml LPS, 5 μg/ml or 10 μg/ml rHSP40, and 10 μg/ml peptidoglycan at 37°C for 24 h, respectively. In the mitogen‐activated protein kinase (MAPK)‐inhibition experiment, DCs were treated with 10 μg/ml rHSP40 and 50 nM SB203580 (CAT# S1076, Selleckchem), 50 nM SP600125 (CAT# 1460, Selleckchem), or 60 nM U0126 (CAT# S1102, Selleckchem).

### 2.7 Knockdown of TLR2 and TLR4 in DCs

The lentiviral vector pLKO.1 (Antihela) was used to overexpress short hairpin RNA (shRNAs) targeting TLR2 and TLR4 (shTLR4 and shTLR4). The primers used to construct pLKO.1‐shTLR2 and pLKO.1‐shTLR4 are listed in Table 1. DCs were seeded into six‐well plates at a density of 3 ×  10^6^ cells per well and then transfected with 4 μg shTLR4 or shTLR4 using Lipofectamine RNAiMAX (CAT# 13778030, Life Technology) at 37°C. Forty‐eight hours after transfection, DCs were treated with 5 μg/ml LPS, 10 μg/ml rHSP40, and 10 μg/ml peptidoglycan (PGN; CAT# S11184, Shanghai Yuanye Bio‐Technology Co).

**Table 1:** Primers for plasmid construction and RT‐qPCR

Primers	Sequence (5′→3′)
IL12A‐QF	ACCTCTTTCATAACTAATGG
IL12A‐QR	ACATCTTCAAGTCTTCATAA
IL12B‐QF	GTTGGTCATCTCTTGGTT
IL12B‐QR	GACATAAACATCTTTCTTCAGT
IL23A‐QF	AGATGAAGAGACTACAAATGAT
IL23A‐QR	AAGCAGAACTGACTGTTG
IL10‐QF	GCCTTTAATAAGCTCCAA
IL10‐QR	TTCGTATCTTCATTGTCAT
IL1B‐QF	CTTCAGCCAATCTTCATT
IL1B‐QR	ATTGCCACTGTAATAAGC
IL6‐QF	GGATTCAATGAGGAGACTT
IL6‐QR	ATCTGTTCTGGAGGTACT
TNFa‐QF	CAACCTCTTCTGGCTCAA
TNFa‐QR	TGGTGGTCTTGTTGCTTA
shTLR2‐F	CCGGGCATCTGATAATGACAGAGTTCTCGAGAACTCTGTCATTATCAGATGCTTTTT
shTLR2‐R	AATTAAAAAGCATCTGATAATGACAGAGTTCTCGAGAACTCTGTCATTATCAGATGC
shTLR4‐F	CCGGCCGCTGGTGTATCTTTGAATACTCGAGTATTCAAAGATACACCAGCGGTTTTT
shTLR4‐R	AATTAAAAACCGCTGGTGTATCTTTGAATACTCGAGTATTCAAAGATACACCAGCGG

Abbreviations: F, forward primer for plasmid construction, QF, forward primer for RT‐qPCR; QR, reverse primer for RT‐qPCR; RT‐qPCR, reverse‐transcription quantitative polymerase chain reaction; R, reverse primer for plasmid construction.

### 2.8 Reverse‐transcription quantitative polymerase chain reaction (RT‐qPCR)

Total RNA was extracted from DCs using Total RNA Extraction Reagent (CAT# R401‐01, Vazyme). HSP40, TNF‐α, IL‐1β, IL‐6, IL‐10, IL‐12p40, IL‐12p35, and IL‐23p19 cDNAs were synthesized using HiScript II Reverse Transcriptase (CAT# R201‐01, Vazyme). qPCR was performed using an iQ5 Real‐Time PCR Detection System (Bio‐Rad Laboratories) with an AceQ qPCR SYBR Green Master Mix Kit (CAT# Q111‐02, Vazyme). Thermocycling conditions were 96°C for 5 min, followed by 50 cycles of 96°C for 15 s, 60°C for 15 s, and 68°C for 15 s. The primers used are listed in Table 1.

### 2.9 Enzyme‐linked immunosorbent assay (ELISA)

The cell supernatant was collected after centrifugation at 1000*g* for 20 min at 4°C. IL‐6 (CAT# D6050, R&D), IL‐10 (CAT# DY217B‐05, R&D), IL‐12p70 (CAT# D1200, R&D), and TNF‐α (CAT# DTA00D, R&D). ELISA kits were used to measure the levels of cytokines secreted by DCs according to the manufacturer's instructions.

### 2.10 Western blot analysis

DCs were seeded in six‐well plates and cultured in Dulbecco's modified eagle medium containing 10% fetal bovine serum for 24 h. Total protein from each group was determined using the BCA quantification method (CAT# E112‐01, Vazyme). Samples (20 μg protein per lane) were loaded on 12% denaturing sodium dodecyl‐sulfate polyacrylamide gel electrophoresis (SDS‐PAGE) gels for electrophoresis and then transferred onto polyvinylidene difluoride membranes. After blocking with 5% BSA for 2 h, the membrane was probed with primary antibodies overnight at 4°C. Subsequently, the membranes were washed with Tris‐HCl buffer, followed by incubation with the corresponding secondary antibodies prepared at 28°C for 1 h. The bands were visualized using chemiluminescence detection reagent (CAT# 32132, ThermoFisher Scientific). Target protein expression levels were quantified using the ImageJ software (NIH). The primary antibodies used were anti‐6× His tag (1:1000, CAT# ab18184, Abcam), anti‐JNK (1:2000, CAT# 24164‐1‐AP, ProteinTech), anti‐p‐JNK (1:2000, CAT# 80024‐1‐RR, ProteinTech), anti‐p38 (1:1000, CAT# 9212, Cell Signaling Technology), anti‐p‐p38 (1:1000, CAT# 9211, Cell Signaling Technology), and anti‐GAPDH (1:3000, CAT# 10494‐1‐AP, ProteinTech). Secondary antibodies are horse radish peroxidase (HRP)‐Goat Anti‐Mouse IgG (H + L) (1:5000, CAT# SA00001‐1, ProteinTech) and HRP‐Goat anti‐Rabbit IgG (H + L) (1:5000, CAT# SA00001‐2, ProteinTech).

### 2.11 Statistical analyses

The data were analyzed using SPSS 22.0 (GraphPad Software). Data are presented as means ± standard deviation (SD). Analysis of variance (ANOVA) followed by Tukey's post hoc test was used for multiple comparisons among the three groups. The Student's *t* test was used to compare the differences between the two groups. Statistical significance was set at *p* < .05.

## 3 RESULTS

### 3.1 Isolation and identification of DC cells

**Figure 1:**
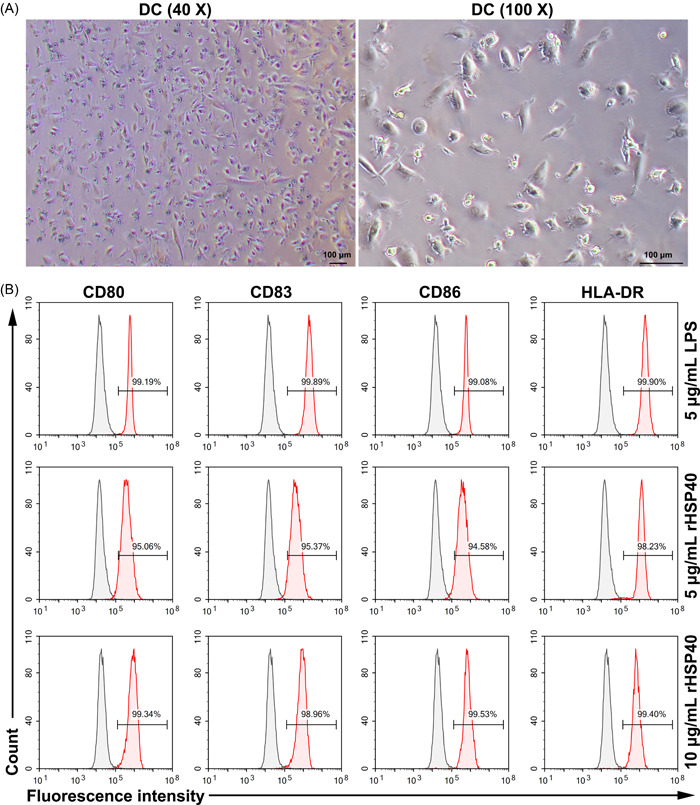
Dendritic cells (DCs) were successfully isolated from human peripheral blood. (A) Representative images of DCs. (B) Markers of mature DCs were detected by flow cytometry. Isolated DCs were stimulated with lipopolysaccharide (LPS) or recombinant HSP40 (rHSP40) for 24 h.

The morphology of isolated DCs was observed (Figure 1A). rHSP40 was successfully expressed and purified (Supporting Information: Figure 1). Then, rHSP40 was used to stimulate the DCs. As shown in Figure 1B, after stimulation with 5 μg/ml LPS, 5 μg/ml rHSP40, or 10 μg/ml rHSP40, DCs underwent maturation and expressed high levels of markers including CD80, CD86, CD83, and HLA‐DR. Moreover, the percentage purity of DCs was more than 98% (Figure 1B).

### 3.2 Activation of DCs increases the levels of inflammatory cytokines

**Figure 2:**
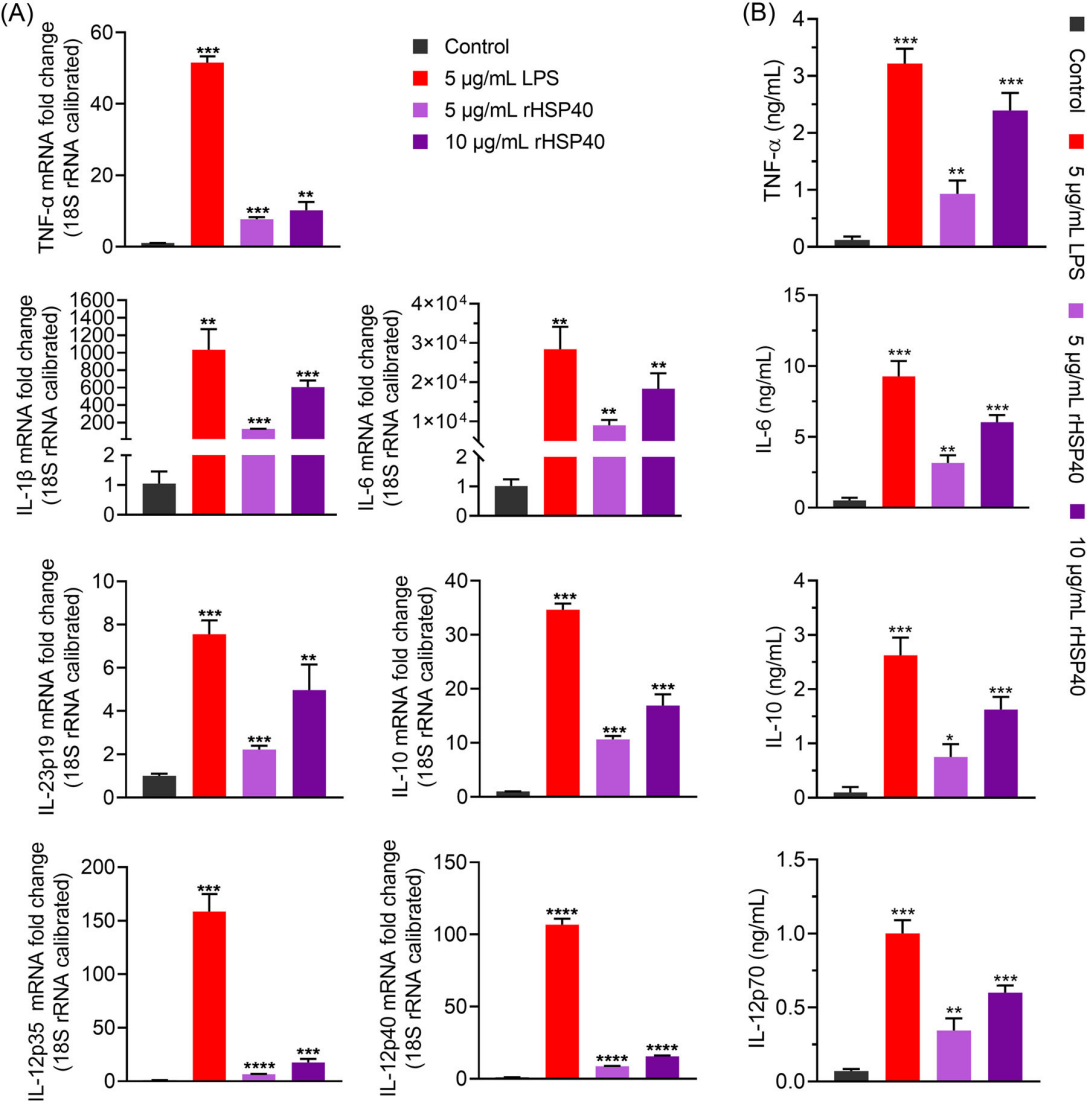
Activation of dendritic cells (DCs) increases the levels of inflammatory cytokines. (A) Messenger RNA (mRNA) levels of tumor necrosis factor‐α (TNF‐α), Interleukin‐1β (IL‐1β), IL‐6, IL‐10, IL‐12p40, IL‐12p35, and IL‐23p19 were determined using reverse‐ transcription quantitative polymerase chain reaction (RT‐qPCR). (B) Enzyme‐linked immunosorbent assay (ELISA) was performed to measure the secretion levels of TNF‐α, IL‐6, IL‐10, and IL‐12p70 in supernatant. DCs were stimulated with lipopolysaccharides (LPS) and recombinant HSP40 (rHSP40) for 24 h, respectively. Analysis of variance (ANOVA) followed by Tukey's post hoc test: *n* = 3, versus group “Control,” *p < .05, **p < .01, ***p < .001.

Cytokine secretion was also evaluated to determine DC activation. As can be seen in Figure 2A, the mRNA levels of IL‐1β, TNF‐α, IL‐6, IL‐10, IL‐23p19, IL‐12p40, and IL‐12p35 were upregulated by LPS and rHSP40. Moreover, the mRNA levels were upregulated in an rHSP40‐concentration‐dependent manner. In addition, the levels of TNF‐α, IL‐6, IL‐10, and IL‐12p70 in the supernatant were also measured. The results of ELISA were consistent with those of RT‐qPCR (Figure 2B). According to these results, 10 μg/ml rHSP40 was chosen in the future work.

**Figure 3:**
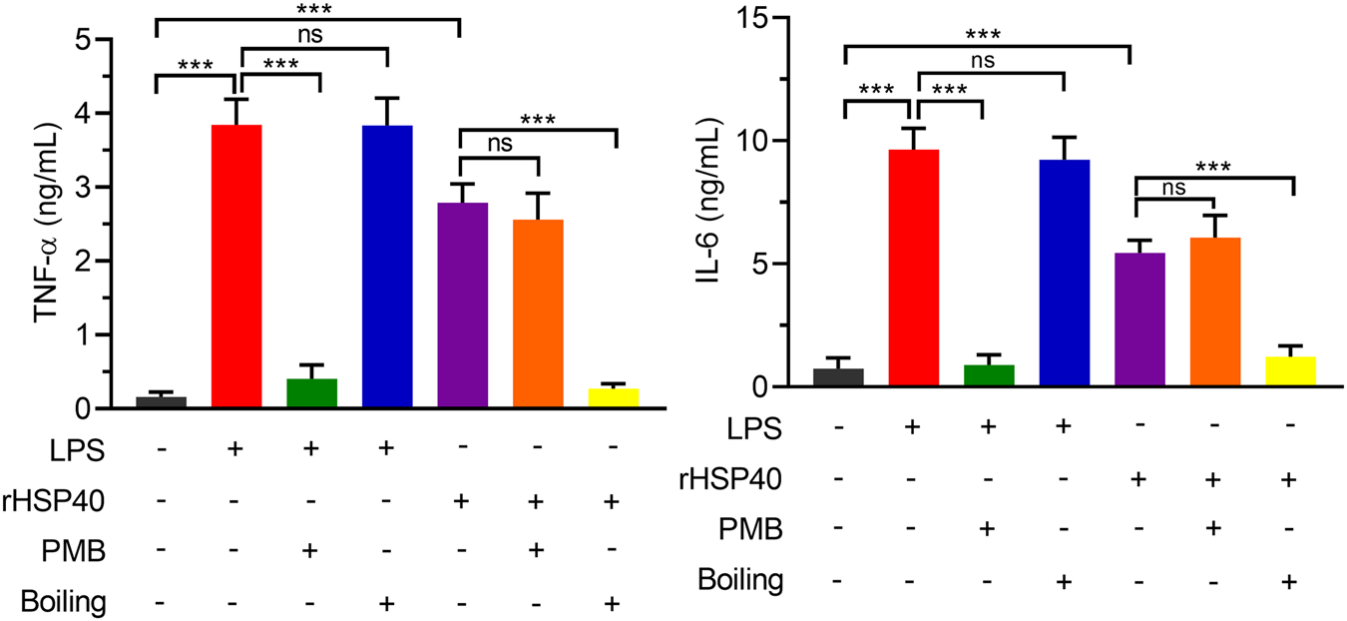
Exclusion of the activation of dendritic cells (DCs) induced by lipopolysaccharides (LPS) contamination. rHSP40 sample was treated with polymyxin B (PMB) or boiled for 30 min before stimulating DCs. LPS was used as a positive control. LPS, 5 μg/ml for 24 h. rHSP40, 10 μg/ml for 24 h. Analysis of variance (ANOVA) followed by Tukey's post hoc test: *n* = 3; ns, no significance; ***p < .001.

Detection of endotoxin in the rHSP40 sample indicated that there were no residual LPS. To further exclude the effect of LPS contamination and verify the stimulating activity of rHSP40, we treated LPS and rHSP40 with polymyxin B (PMB) or boiled LPS and rHSP40 before using them to stimulate DCs. As shown in Figure 3, LPS did not induce the secretion of IL‐6 and TNF‐α after treatment with PMB, in contrast to rHSP40. Moreover, boiling eliminated the rHSP40‐induced secretion of TNF‐α and IL‐6.

### 3.3 The activation of DCs by HSP40 is TLR4‐dependent

**Figure 4:**
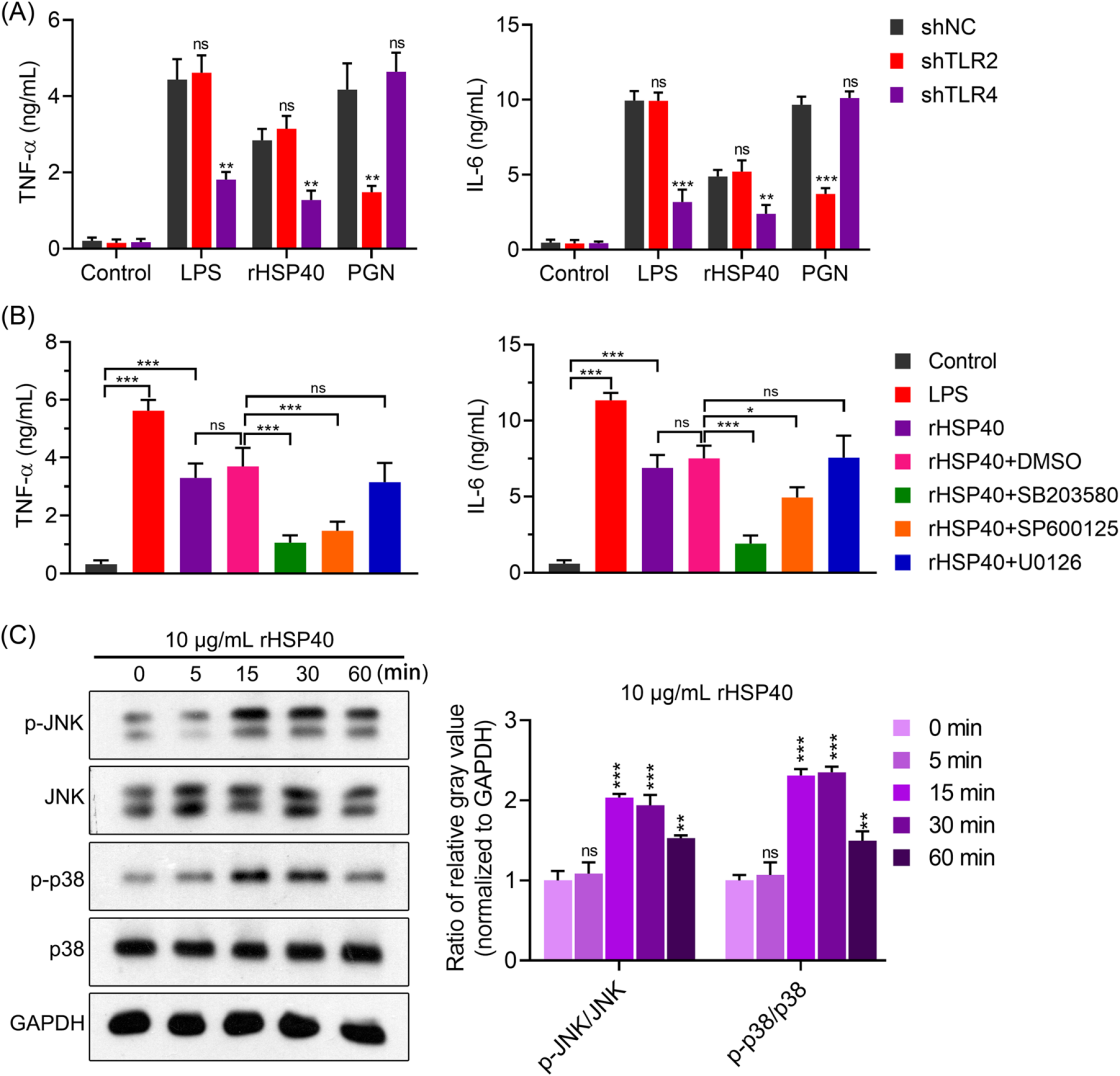
Recombinant HSP40 (rHPS40) activated dendritic cells (DCs) via toll‐like receptor 4 (TLR4)‐dependent p38 mitogen‐activated protein kinase (p38 MAPK) and c‐Jun N‐terminal kinase (JNK) signaling pathways. (A) The activation of DCs by rHSP40 was TLR4‐dependent. PGN, peptidoglycan. Analysis of variance (ANOVA) was followed by Tukey's post hoc test: *n* = 3, versus group “shNC,” ns no significance, **p < .01, ***p < .001. (B) The activation of DCs by rHSP40 was inhibited by p38 MAPK and JNK inhibitors. ANOVA followed by Tukey's post hoc test: *n* = 3, ns, no significance; *p < .05, ***p < .001. (C) rHSP40 treatment increased phosphorylated levels of p38 and JNK. Left panel: Representative image of western blot analysis. Right panel: Statistical quantification based on the optical intensity of bands. ANOVA followed by Tukey's post hoc test: *n* = 3, versus group “0 min,” ns, no significance, **p < .01, ***p < .001.

To confirm whether the activation of DCs is TLR2‐ or TLR4‐dependent, we knocked down TLR2 or TLR4 in DCs and then treated them with the TLR2 agonist PGN and TLR4 agonist LPS, respectively. As shown in Figure 4A, TLR2 deficiency significantly suppressed PGN‐induced secretion of IL‐6 and TNF‐α. TLR4 knockdown inhibited both LPS‐induced and rHSP40‐induced secretion of IL‐6 and TNF‐α.

### 3.4 HSP40 activates DCs via p38 MAPK and JNK signaling pathways

We further investigated the activation of pathways downstream of TLR4. As shown in Figure 4B, the p38 MAPK inhibitor SB203580 and c‐Jun N‐terminal kinase (JNK) inhibitor SP600125 significantly suppressed the rHSP40‐induced secretion of IL‐6 and TNF‐α. However, the ERK inhibitor U0126 did not eliminate the effects of rHSP40. Moreover, western blot analysis showed that the phosphorylated levels of p38 MAPK and JNK in DCs increased after rHSP40 treatment. The peak value was present at 15–30 min and then decreased (Figure 4C).

## 4 DISCUSSION

As the most important professional APCs in the body, DCs play an important role in the immune response. Mature DCs present antigens to initial CD4^+^ T cells, and activated DCs express costimulatory molecules and cytokines to induce the polarization of initial CD4^+^ T cells to Th1, Th2, and Th17 cells,^32^ thus causing the host to produce the corresponding adaptive immune response. HSP was found to induce DC maturation and enhance the antigen presentation ability of DCs.^33^, ^34^ Our results revealed that HSP40 successfully activated human DCs to express costimulatory molecules CD80/CD86 and secrete inflammatory cytokines including TNF‐α, IL‐6, IL‐10, and IL‐12.

After entering the body, *S. pneumoniae* is recognized by the pattern recognition receptors (PRRs) of APCs. TLR2 and TLR4 are PRRs that are highly related to *S. pneumoniae*. TLR2 recognizes the peptidoglycans of *S. pneumoniae*. TLR4 recognizes lipoteichoic acids derived from *S. pneumoniae*.^35^ Studies have shown that TLRs can recognize HSP to initiate an intracellular signaling cascade.^20^, ^36^, ^37^ In this study, TLR4 knockdown, other than TLR2 knockdown, alleviated the rHSP40‐induced cytokine secretion, which suggests that HSP40 stimulates human DCs through recognization of TLR4. The binding of exogenous ligands to TLR4 usually triggers MAPK signaling pathways.^10^, ^23^ Our study demonstrated that HSP40 activates human DCs via p38 MAPK and JNK signaling pathways, which is consistent with the study of Wu et al. in mice. The levels of p‐p38 and p‐JNK began to decrease after activation of DCs with HSP40 at 60 min, indicating that the stimulation of MAPK signaling may achieve a peak and then attenuate.

There are several limitations to this study. We need to confirm the reorganization of HSP40 by TLR4 using an optical surface plasmon resonance bioanalyzer such as Biacore. Moreover, our conclusions should be validated by animal experiments. In addition, without target population, the calculation (Power Analysis) and justification of the sample size were not done. In the future, we should also reveal the mechanism by which HSP40 activates human macrophages.

## 5 CONCLUSION

This study, for the first time, confirmed that HSP40 activates human DCs via TLR4‐dependent p38 MAPK and JNK signaling pathways.

## AUTHOR CONTRIBUTIONS

Li‐Na Chen conceived and designed the study. Jing‐jing Liu and Jian‐Cheng Lin performed the experiments and collected the data. Li‐na Chen analyzed the data. Li‐na Chen and Jing‐jing Liu wrote the manuscript. All authors approved the manuscript for publication.

## CONFLICT OF INTEREST

All authors declare no conflict of interest.

## Supporting information

Supporting information.

## Data Availability

All data in the present study are available from the corresponding author on reasonable request.
