# Supplementary material for: Heat shock protein 40 of Streptococcus pneumoniae induces immune response of human dendritic cells via TLR4‐dependent p38 MAPK and JNK signaling pathways
Source: Immun Inflamm Dis. 2022 Nov 25;10(12):e735. doi: 10.1002/iid3.735 (PMC9695094; doi:10.1002/iid3.735)
Supplement: Supplementary file 1 — Supporting information. [file IID3-10-e735-s001.docx]

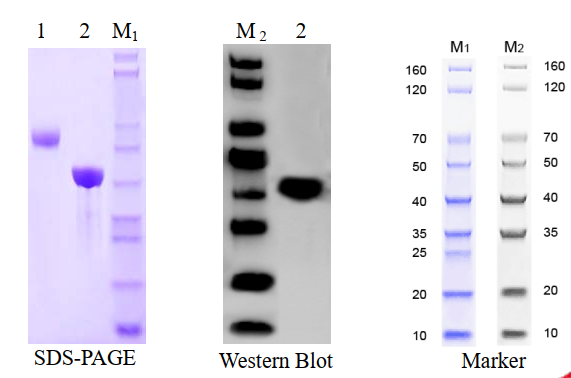


Supplementary figure 1. rHSP40 was expressed and purified. Lane 1: 2.0 μg BSA; Lane 2: 4.0 μg rHSP40; Lane M1: SDS-PAGE marker; Lane M2: western blotting marker. Anti-His antibody was used.
